# Supplementary material for: Cardiopulmonary Bypass-Induced IL-17A Aggravates Caspase-12-Dependent Neuronal Apoptosis Through the Act1-IRE1-JNK1 Pathway
Source: Biomolecules. 2025 Aug 6;15(8):1134. doi: 10.3390/biom15081134 (PMC12384059; doi:10.3390/biom15081134)
Supplement: Supplementary file 1 [file biomolecules-15-01134-s001.zip › biomolecules-3711573-supplementary.pdf]

Figure S1: Typical WB bands of Caspase-3 in four brain regions

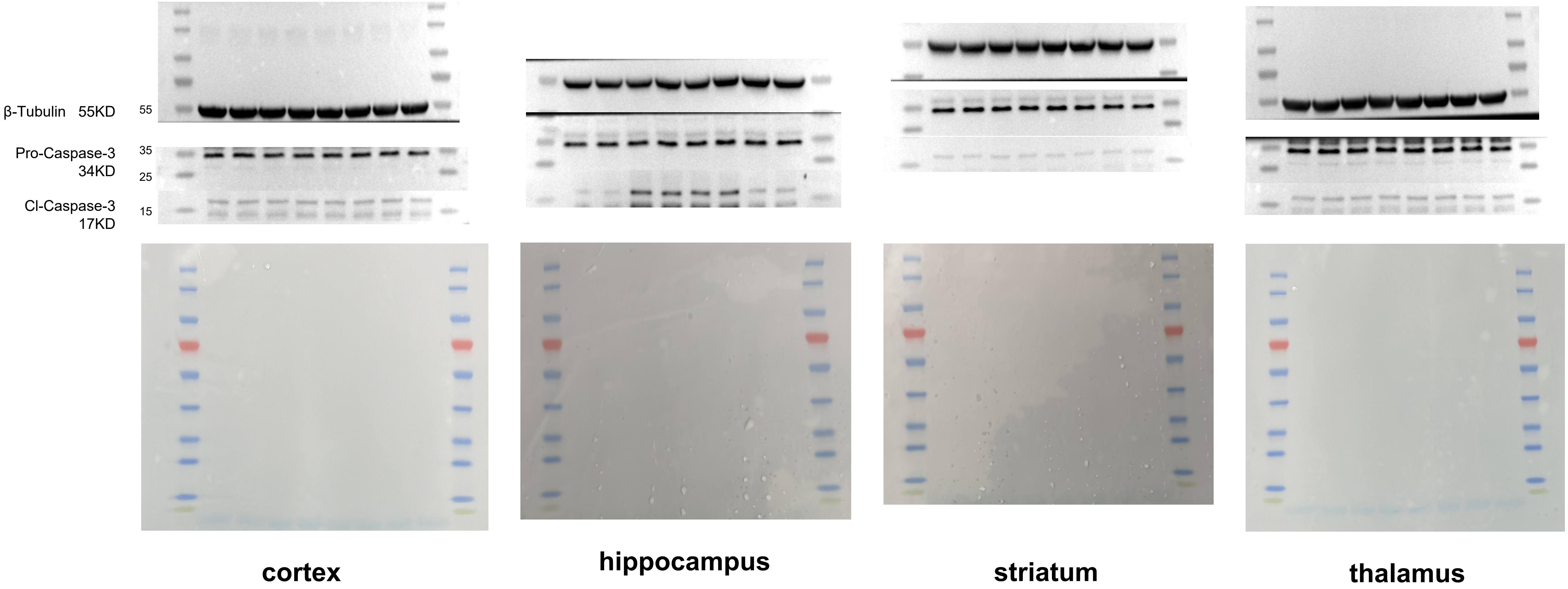

Figure S2: IL-17RA membrane translocation

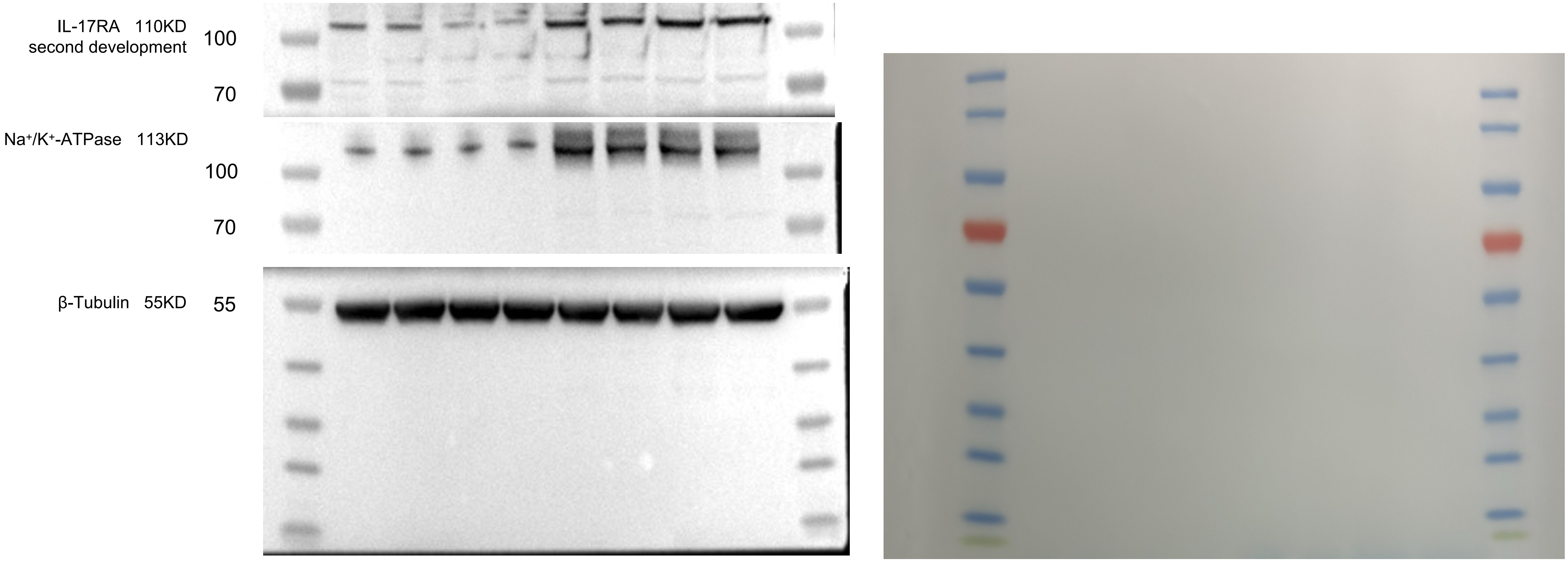

Figure S3: Typical proteins on different apoptosis pathways

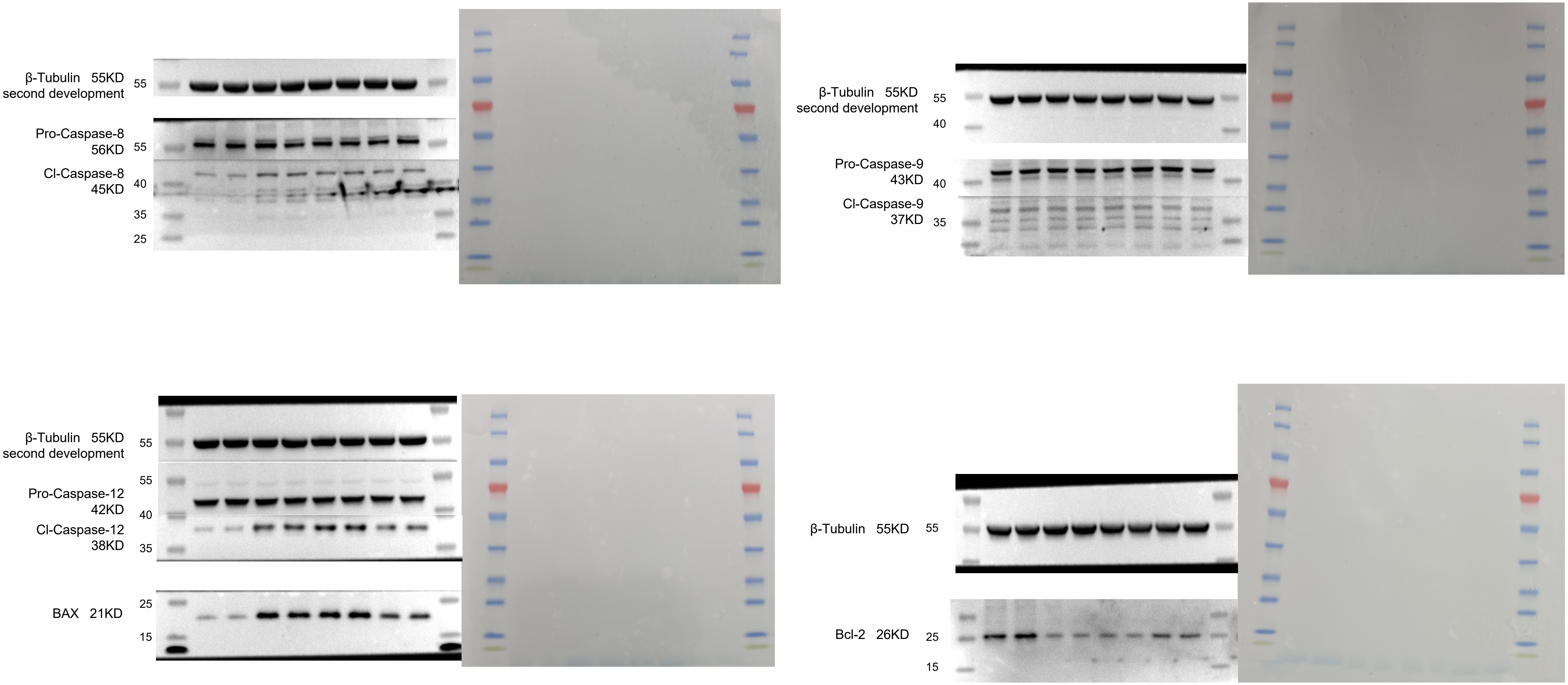

Figure S4: Typical WB bands of ERS-related proteins and Co-IP

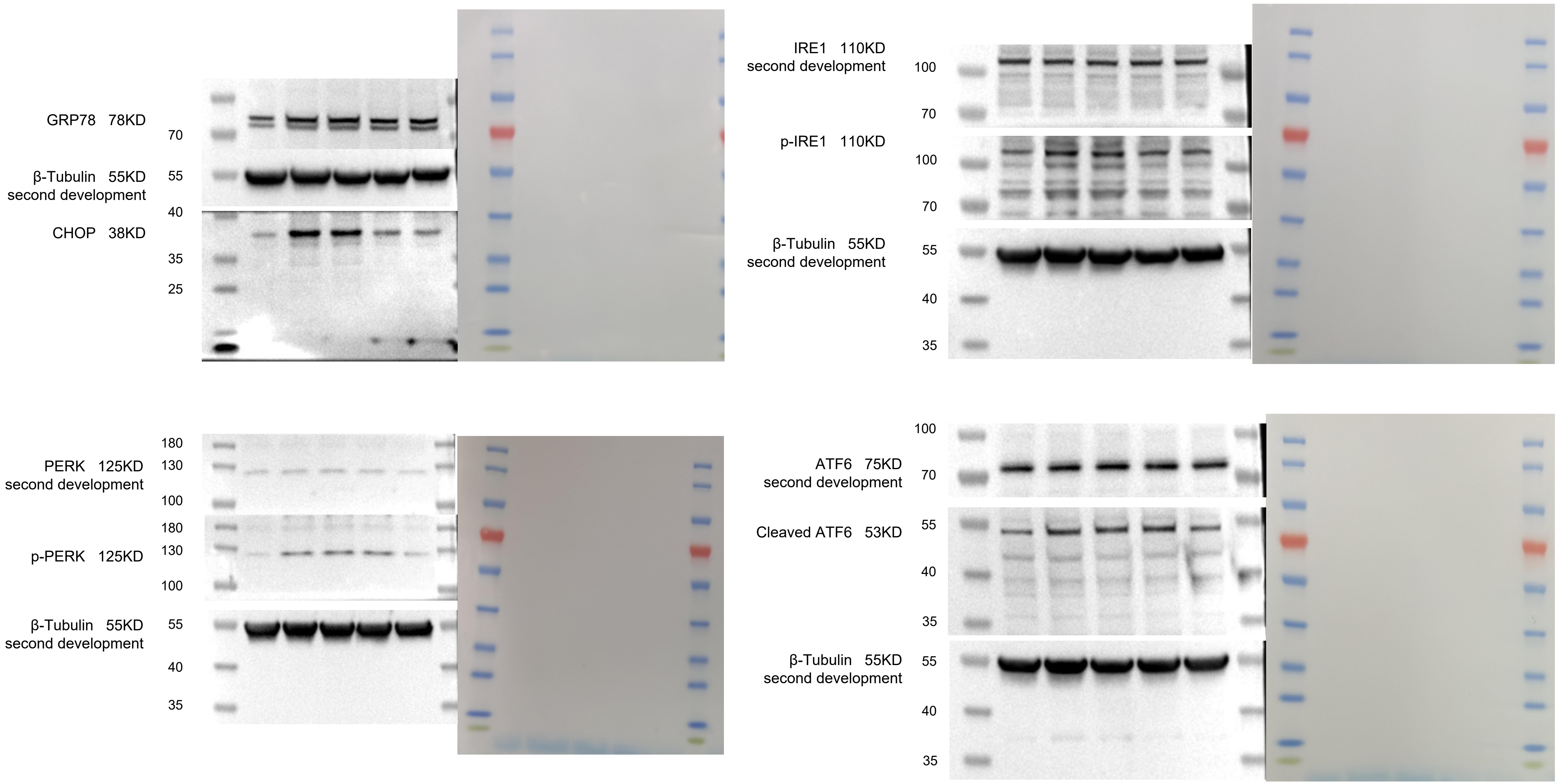

Figure S5: Typical WB bands of ERS-related proteins and Co-IP at animal level

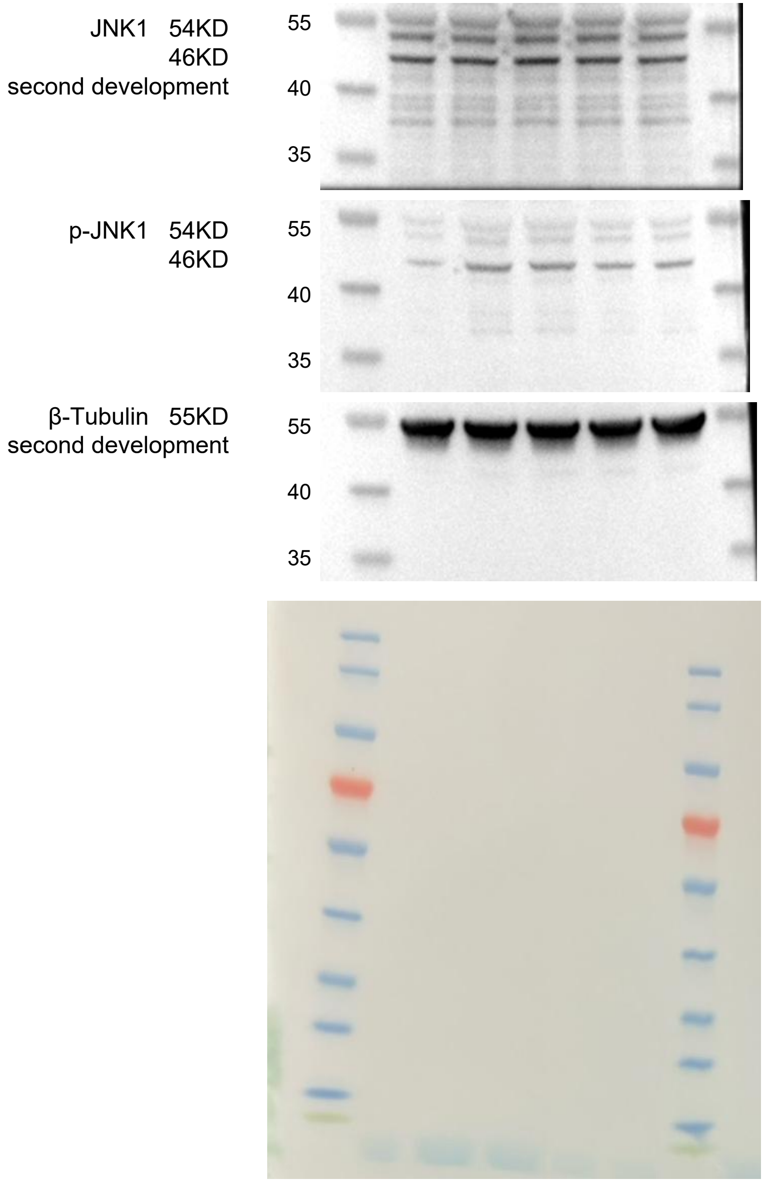

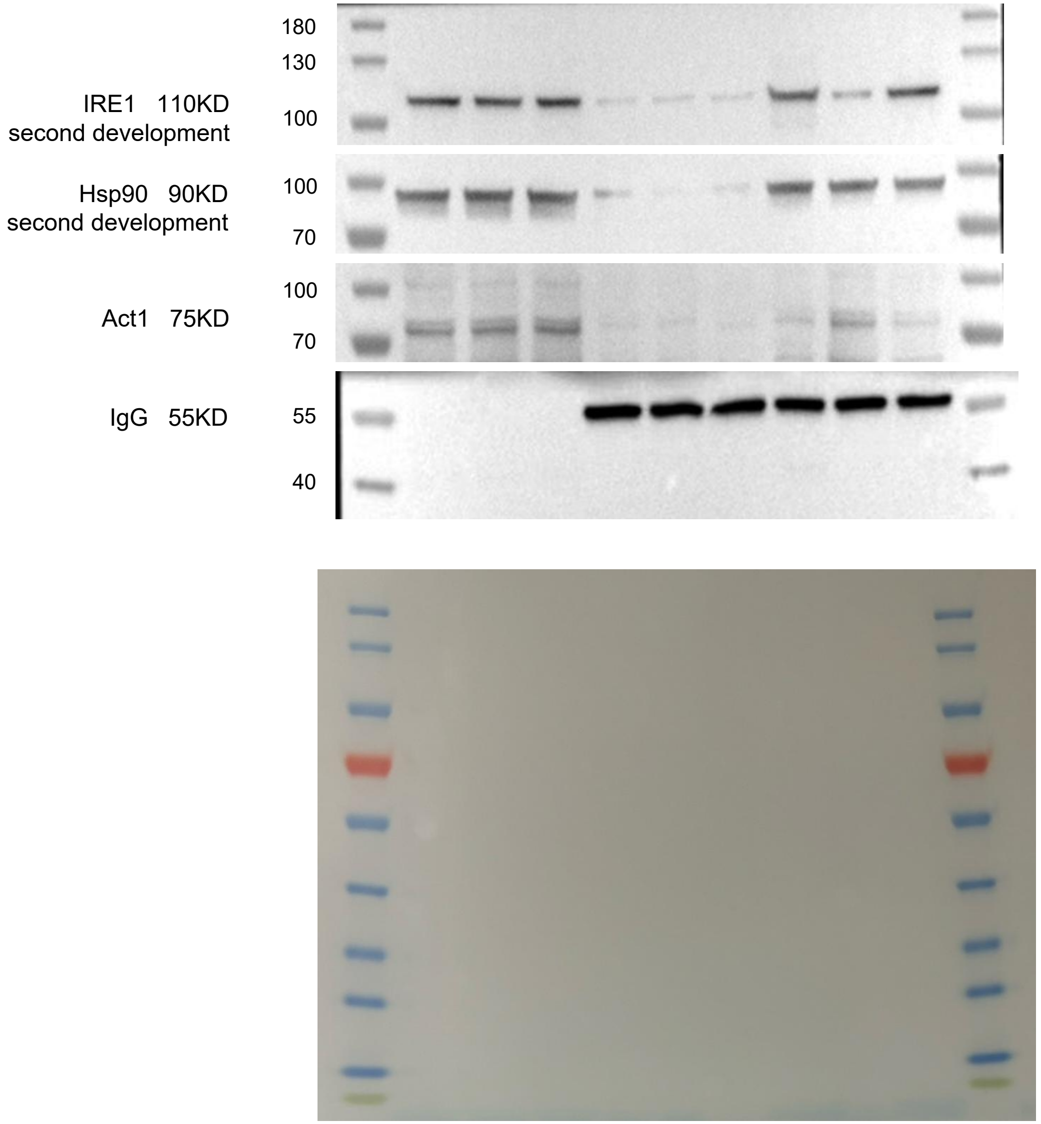

Figure S6: Typical WB bands of apoptosis- and ERS-related proteins at cell level; Co-IP at cell level

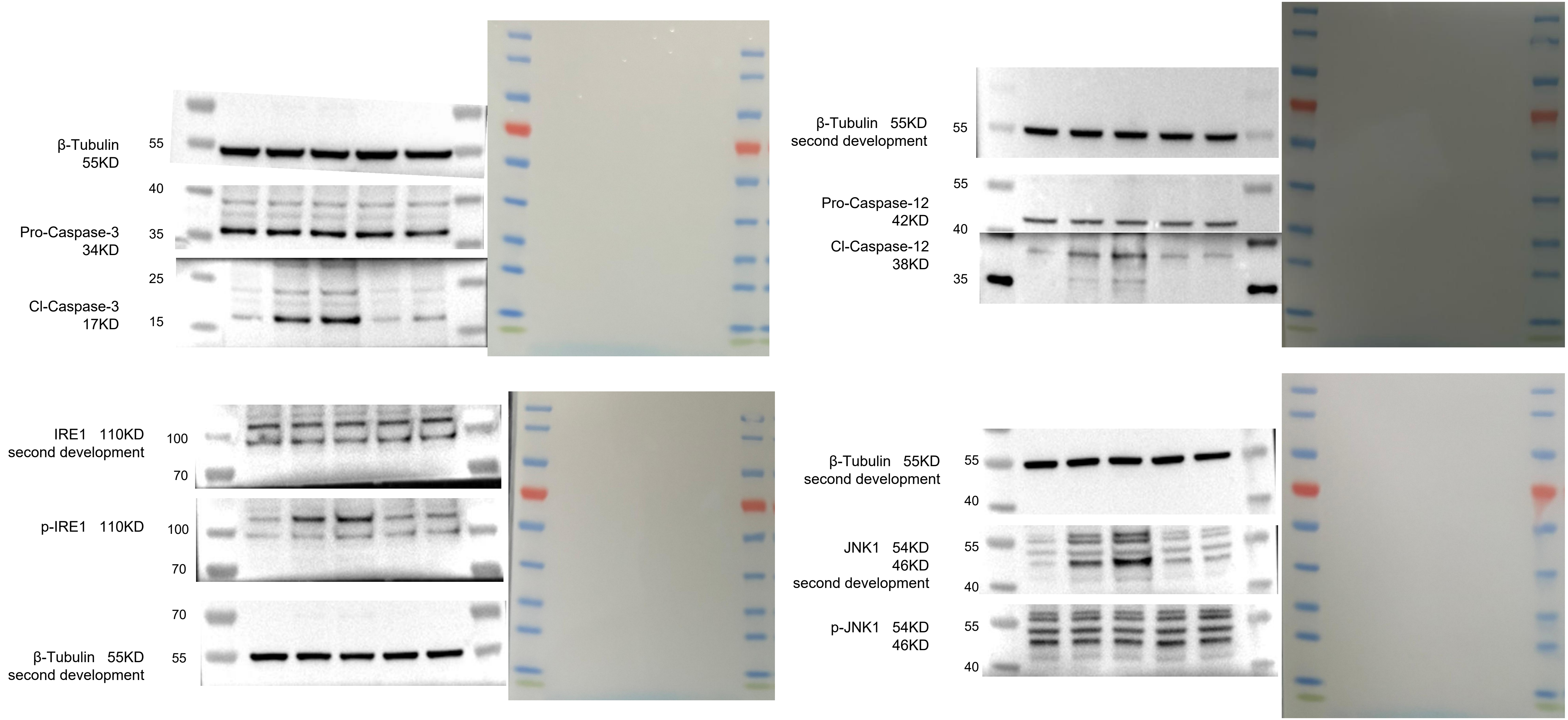

Figure S6: Typical WB bands of apoptosis- and ERS-related proteins at cell level;
Co-IP at cell level

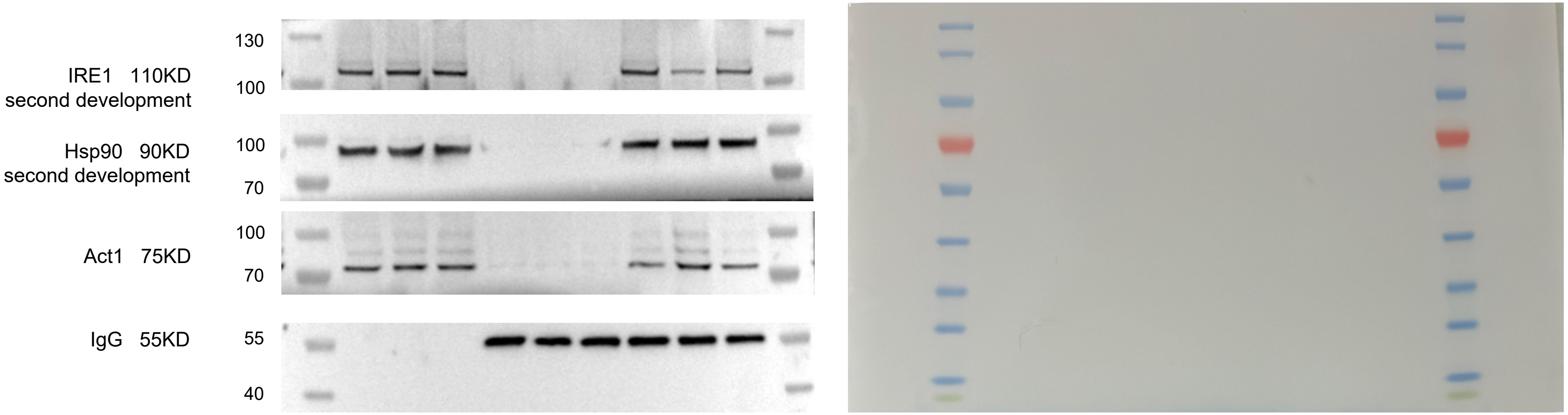

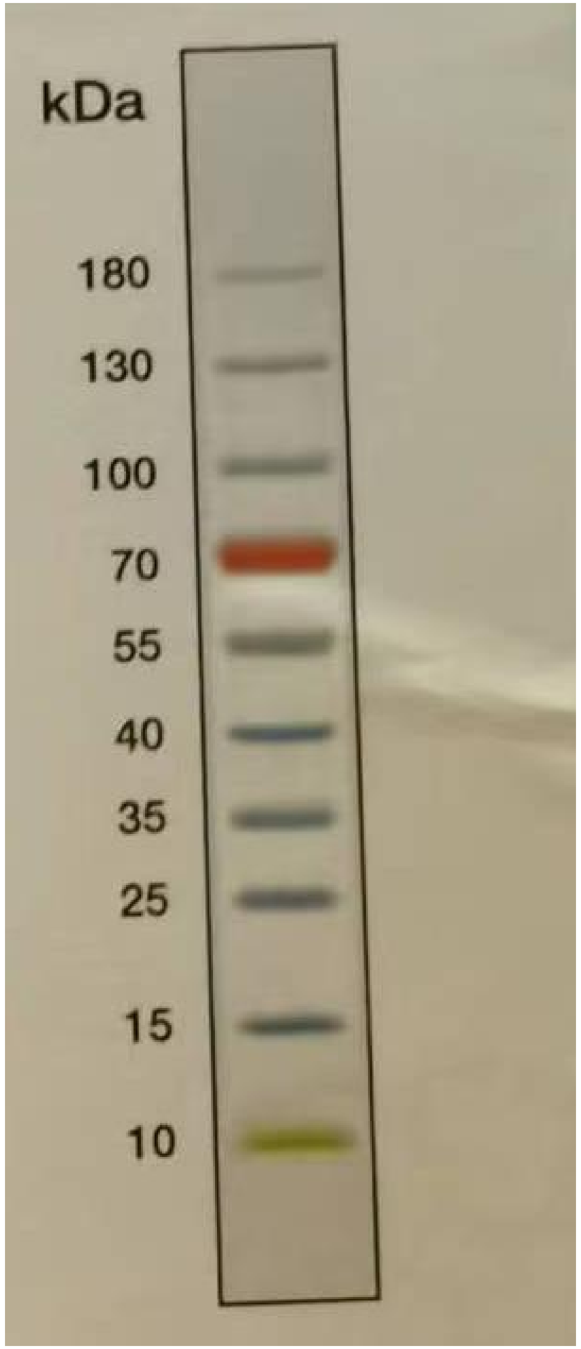
kDa
180
130
100
70
55
40
35
25
15
10
